# Prevalence, Patterns, and Predictors of Hearing Handicap in Relation to Personal Listening Device Use: A Cross-Sectional Study in Saudi Arabia

**DOI:** 10.3390/healthcare14162482

**Published:** 2026-08-11

**Authors:** Dhiyaa A. H. Otayf, Albaraa H. Hakami, Saja A. Almraysi, Mohammad A. Jareebi, Saud N. Alwadani, Khalid I. Hakami, Waleed A. Alabdali, Yazan A. Bajawi, Abdullah Mawkili, Tawfiq Khurayzi, Mashael Solan Mahnashi, Raghad Wasil AlNahwe, Farjah H. Algahtani, Amal J. Alfaifi, Ghazi I. Al Jowf

**Affiliations:** 1 Faculty of Medicine, Jazan University, Jazan 45142, Saudi Arabia; baraa7ake@gmail.com (A.H.H.); sajaalasiri1@gmail.com (S.A.A.); saudwadani78@gmail.com (S.N.A.); hakamikhalid23@gmail.com (K.I.H.); dr.waleedalabdali@gmail.com (W.A.A.); yz.bajawi@gmail.com (Y.A.B.); 2 Department of Family and Community Medicine, College of Medicine, Jazan University, Jazan 45142, Saudi Arabia; 3 Department of Surgery, College of Medicine, Jazan University, Jazan 45142, Saudi Arabia; mawkili@hotmail.com; 4 Cochlear Implant Center, King Fahad Central Hospital, Jazan Health Cluster, Jazan 82666, Saudi Arabia; tawfiqaakk@hotmail.com; 5 King Abdullah Ear Specialist Center (KAESC), King Saud University Medical City, King Saud University, Riyadh 12373, Saudi Arabia; 6 Department of Otolaryngology–Head and Neck Surgery, King Saud University Medical City, King Saud University, Riyadh 12373, Saudi Arabia; mashael2468m@gmail.com (M.S.M.); alnahwe.raghad@gmail.com (R.W.A.); 7 Oncology Center, Chair of Epidemiology and Public Health Research, Faculty of Medicine, King Saud University Medical City, King Saud University, Riyadh 12373, Saudi Arabia; falgahtani@ksu.edu.sa; 8 Department of Family Medicine, Jazan Health Cluster, Jazan 45142, Saudi Arabia; ajh6666@hotmail.com; 9 Department of Public Health, College of Applied Medical Sciences, University Medical Clinics Complex, King Faisal University, Al Hofuf 37912, Saudi Arabia; galjowf@kfu.edu.sa

**Keywords:** personal listening devices, earphones, headphones, hearing handicap, Revised Hearing Handicap Inventory, noise-induced hearing loss, safe listening, Saudi Arabia, cross-sectional study

## Abstract

**Background/Objectives**: Personal listening devices (PLDs) have become ubiquitous in modern daily life, raising growing concerns about their cumulative impact on auditory health. This study examined the prevalence and patterns of PLD use and the independent predictors of perceived hearing handicap among Saudi adults. **Methods**: A nationwide cross-sectional survey was conducted between November 2025 and January 2026 among 2018 adults (aged 18 years or older) residing in Saudi Arabia. Participants completed a self-administered online questionnaire assessing sociodemographic characteristics, PLD usage patterns, and hearing handicap measured by the Revised Hearing Handicap Inventory (RHHI-18). **Results**: Participants had a mean age of 29 ± 12 years, 54.0% were female, and 93.9% were Saudi nationals. Earphones and headphones were used by 51.1% and 47.7% of participants, respectively, and 68.1% reported current use of at least one personal listening device. The mean RHHI-18 total score was 10 ± 18, and 23.0% of participants scored within the hearing handicap range (mild-to-moderate: 16.2%; significant: 6.8%). All PLD use categories were independently associated with higher RHHI-18 scores relative to non-use: headphones only (beta = 4.58; *p* < 0.001), earphones only (beta = 3.29; *p* = 0.002), and combined use (beta = 2.87; *p* = 0.005). Further independent correlates of higher RHHI-18 scores were stroke (beta = 21.51; *p* < 0.001), diabetes mellitus (beta = 8.64; *p* < 0.001), hypertension (beta = 4.84; *p* = 0.006), non-Saudi nationality (beta = 3.92; *p* = 0.014), male sex (beta = 3.09; *p* < 0.001), and older age (beta = 0.16; *p* = 0.002). **Conclusions**: PLD use is highly prevalent among Saudi adults and is independently associated with greater perceived hearing handicap. These findings support public health efforts promoting safe listening practices at the population level.

## 1. Introduction

Hearing is one of the most fundamental human senses, underpinning communication, social participation, and cognitive development. A clinically important distinction exists between hearing impairment and hearing handicap: while impairment denotes structural or functional loss within the auditory system, handicap describes the broader social, vocational, and psychological disadvantages that arise when an individual is unable to perform activities of daily living [1]. Globally, disabling hearing loss has reached crisis proportions. More than 1.5 billion people are currently affected by some degree of auditory loss, a figure projected to rise to 2.5 billion by 2050 [2]. The consequences extend far beyond the clinical domain; untreated hearing loss incurs an estimated annual economic burden of approximately 980 billion USD worldwide, driven by healthcare expenditure, lost workforce productivity, and educational exclusion, particularly in low- and middle-income countries where access to assistive technology remains below 17% [2,3].

In the Arabian Peninsula and broader Middle East, hearing handicap carries an additional epidemiological dimension shaped by genetic and environmental factors. Congenital sensorineural hearing loss is disproportionately prevalent in the Kingdom of Saudi Arabia, predominantly inherited as an autosomal recessive trait potentiated by high rates of consanguineous marriage [4]. In response, the Saudi healthcare system has introduced the Universal Newborn Hearing Screening (UNHS) Programme under Vision 2030, prioritizing early identification and intervention [5]. Despite these advances, structural barriers persist, for instance, social stigma surrounding hearing aid use and a scarcity of specialized rehabilitation services in rural provinces continue to convert manageable impairments into lifelong handicaps [4], while the perinatal focus of these programmes leaves acquired, behaviorally mediated hearing risk in adulthood largely unaddressed. Beyond congenital and age-related etiologies, behavioral risk factors have emerged as an increasingly important contributor to hearing handicap, particularly among younger populations. Personal listening devices (PLDs), encompassing earphones and headphones, have become deeply embedded in contemporary daily routines, including studying, commuting, exercising, and entertainment. Prolonged and high-volume exposure through these devices has been linked not only to measurable audiometric hearing loss but also to early functional deficits such as diminished speech clarity, listening fatigue, and difficulty understanding speech in noise [6,7]. These subtle changes frequently precede clinically detectable hearing loss yet often go unrecognized by users.

Despite growing international concern, the evidence base on PLD-related hearing outcomes remains heterogeneous. Studies conducted among medical students in India and the Qassim region of Saudi Arabia have confirmed widespread PLD uptake and associated otological complaints [8,9], while research from South Korea has demonstrated a dose–response relationship between earphone exposure and hearing loss risk among adolescents [6]. However, a 2026 Saudi comparative study found that insert earphone users exhibited higher rates of localized ear pathology, including external ear canal inflammation, cerumen impaction, and ear pain, compared with headphone users [10], contrasting with findings that over-ear headphones may confer greater functional auditory deficits due to higher sustained acoustic energy delivery. Furthermore, most existing studies have relied on objective audiometric measures of hearing loss, with relatively little attention paid to subjective hearing handicap, which more accurately reflects the real-world functional burden experienced by individuals [11]. Within Saudi Arabia specifically, nationally representative data linking PLD use to validated measures of perceived hearing handicap remain absent.

This study addresses this evidence gap by conducting a nationwide cross-sectional assessment of PLD use and its association with hearing handicap among adults in Saudi Arabia. Using the Revised Hearing Handicap Inventory (RHHI-18) as the primary outcome measure, this study integrates demographic, clinical, and behavioral predictors to provide a comprehensive multidimensional profile of PLD-related hearing health in the Kingdom. The findings aim to inform public health policy, clinical screening protocols, and targeted educational interventions within the Saudi context.

## 2. Materials and Methods

### 2.1. Study Design, Setting, and Sample Size

This cross-sectional study was conducted in the Kingdom of Saudi Arabia, targeting adults (≥18 years) residing in the country. Participants were recruited using a convenience sampling technique. Individuals younger than 18 years or those who failed to provide informed consent were excluded from the study. Participants reporting no device use were retained as the reference group for all comparative analyses.

Sample size was estimated using the following formula for proportions:

*n*_0_ = *Z*^2^ · *p* · (1 − *p*)/*e*^2^

A confidence level (*Z*) of 1.96 (corresponding to 95%) was adopted in line with standard practice. The expected proportion (*p*) of hearing handicap prevalence was conservatively set at 0.50, which yielded the maximum possible sample size given the limited evidence on hearing handicap burden in Saudi Arabia. A margin of error (*e*) of 2.19% was applied to enhance estimation precision and capture variability within the diverse Saudi population. The calculated minimum sample size was 2000 participants; the final collected sample comprised 2018 participants.

### 2.2. Data Collection Tool

The data collection tool was developed following a systematic and rigorous process. Items were generated after an extensive literature review and consultation with experts in otology, epidemiology, and public health. A preliminary draft was piloted with a convenience sample of 30 individuals from diverse age, educational, and cultural backgrounds, and items were refined based on participant feedback to enhance clarity and comprehensibility. The final instrument was administered as an online survey via Google Forms.

The instrument comprised three sections. The first captured sociodemographic data including age, weight, height, sex, nationality, residence, region, marital status, educational level, monthly household income, medical and otologic history, physical activity level, and smoking status. The second section assessed audio listening device usage, covering types of devices used and the most frequently used device. The third and final section assessed perceived hearing handicap.

Hearing handicap was measured using the Revised Hearing Handicap Inventory (RHHI-18), a validated self-report questionnaire developed by Cassarly et al. as a psychometrically refined update of the original Hearing Handicap Inventory for Adults and Elderly (HHIA/HHIE) [12]. The RHHI-18 was developed and validated using modern item response theory methods, forming a strong, unidimensional scale of self-perceived hearing handicap with demonstrated screening accuracy against audiometric criteria. The scale comprises 18 items across emotional and social/situational domains. Each item is rated on a 3-point scale (Yes = 4, Sometimes = 2, No = 0), yielding a total score from 0 to 72, with higher scores indicating greater perceived hearing handicap [12].

The questionnaire was administered in Arabic. The RHHI-18 was translated from English into Arabic following the core steps of the ISPOR principles of good practice for the translation and cultural adaptation of patient-reported outcome measures. Two bilingual translators, native Arabic speakers fluent in English, produced independent forward translations, which were reconciled by the research team into a single Arabic version. One independent translator, blinded to the source instrument, performed the back-translation, and the back-translation was compared with the original to verify conceptual and semantic equivalence. Remaining discrepancies were resolved by an expert panel with expertise in otology, epidemiology, and public health. The reconciled Arabic version then underwent cognitive debriefing within the pilot described above, in which 30 participants completed the questionnaire and were asked about the clarity, comprehensibility, and cultural appropriateness of each item; wording was refined accordingly, and the final version was proofread before release.

### 2.3. Data Collection Process

Data collection was conducted between November 2025 and January 2026 among 2018 adults (aged 18 years or older) residing in Saudi Arabia, recruited by convenience sampling through online platforms, including WhatsApp, X (formerly Twitter), Snapchat, Instagram, Telegram, and Facebook, by trained data collectors, to maximize outreach and ensure participation from across all regions of Saudi Arabia. The survey included an introductory section describing study objectives and participants’ rights. Responses were reviewed periodically to identify inconsistencies, and incomplete questionnaires were excluded from the analysis.

The survey was distributed as an open, forwardable link to a questionnaire hosted on Google Forms, shared through public accounts, existing group chats, and personal status updates. No contact lists were compiled or retained for the purposes of the study, and no name, telephone number, national identity number, email address, or IP address was requested or recorded. The introductory page described the study objectives, the voluntary nature of participation, and the right to withdraw at any time before submission, and consent was indicated by proceeding to the questionnaire.

### 2.4. Statistical Analysis

Data were entered into Microsoft Excel (Microsoft Corporation, Redmond, WA, USA) for cleaning and verification prior to analysis. Statistical analyses were performed using RStudio (version 4.2.3; R Foundation for Statistical Computing, Vienna, Austria). Descriptive statistics, including means, standard deviations (SD), and frequencies with percentages, were used to characterize the study sample. Multiple linear regression models were constructed to identify independent predictors of the RHHI-18 total score. Overall model fit was assessed using the coefficient of determination (R^2^), the Akaike information criterion (AIC), the omnibus F-test, and a likelihood-ratio test against an intercept-only model, and the contribution of individual predictors was examined by Type II analysis of variance. All statistical tests were two-tailed, and the significance threshold was set at α = 0.05. Results are reported with 95% confidence intervals (CIs). This study adhered to the Strengthening the Reporting of Observational Studies in Epidemiology (STROBE) guidelines.

### 2.5. Ethical Approval

This study was approved by the Standing Committee for Scientific Research at Jazan University (Reference No. REC-46/12/1549, dated 30 May 2025). All procedures complied with the ethical standards of institutional and national research committees and with the principles of the 1964 Declaration of Helsinki and its subsequent amendments. Informed consent was obtained from all participants prior to data collection. The approved protocol covered anonymous, self-administered online data collection with distribution of the survey link through social media and instant-messaging applications, including WhatsApp.

## 3. Results

### 3.1. Sociodemographic Characteristics

The sociodemographic characteristics of the 2018 study participants are presented in Table 1. The mean age was 29 ± 12 years and the mean BMI was 24 ± 5.9 kg/m^2^. Females constituted the majority of the sample (n = 1089; 54.0%), and most participants were Saudi nationals (n = 1894; 93.9%) residing in urban areas (n = 1634; 81.0%). The majority were single (n = 1344; 66.6%), and most held a bachelor’s degree or diploma (n = 1375; 68.1%). Monthly household income was broadly distributed across four categories; the largest proportion reported incomes above 15,000 SAR (approximately >$4000 USD; n = 568; 28.1%), while a similar proportion reported incomes below 5000 SAR (approximately <$1333 USD; n = 553; 27.4%).

### 3.2. Health Conditions and Lifestyle Characteristics

Health conditions and lifestyle characteristics are summarized in Table 2. Acoustic trauma was the most frequently reported condition (n = 258; 12.8%), followed by a history of ear procedures (n = 240; 11.9%) and hyperlipidemia (n = 180; 8.9%). Diabetes mellitus (n = 173; 8.6%), migraine (n = 145; 7.2%), and hypertension (n = 142; 7.0%) were also notable. Regarding physical activity, 41.8% (n = 844) of participants reported engaging in at least 30 min of activity five days per week, while 39.7% (n = 802) reported no physical activity. Most participants were never smokers (n = 1647; 81.6%), with 10.2% (n = 206) being current smokers and 8.2% (n = 165) ex-smokers.

### 3.3. Audio Listening Device Usage Patterns

**Figure 1:**
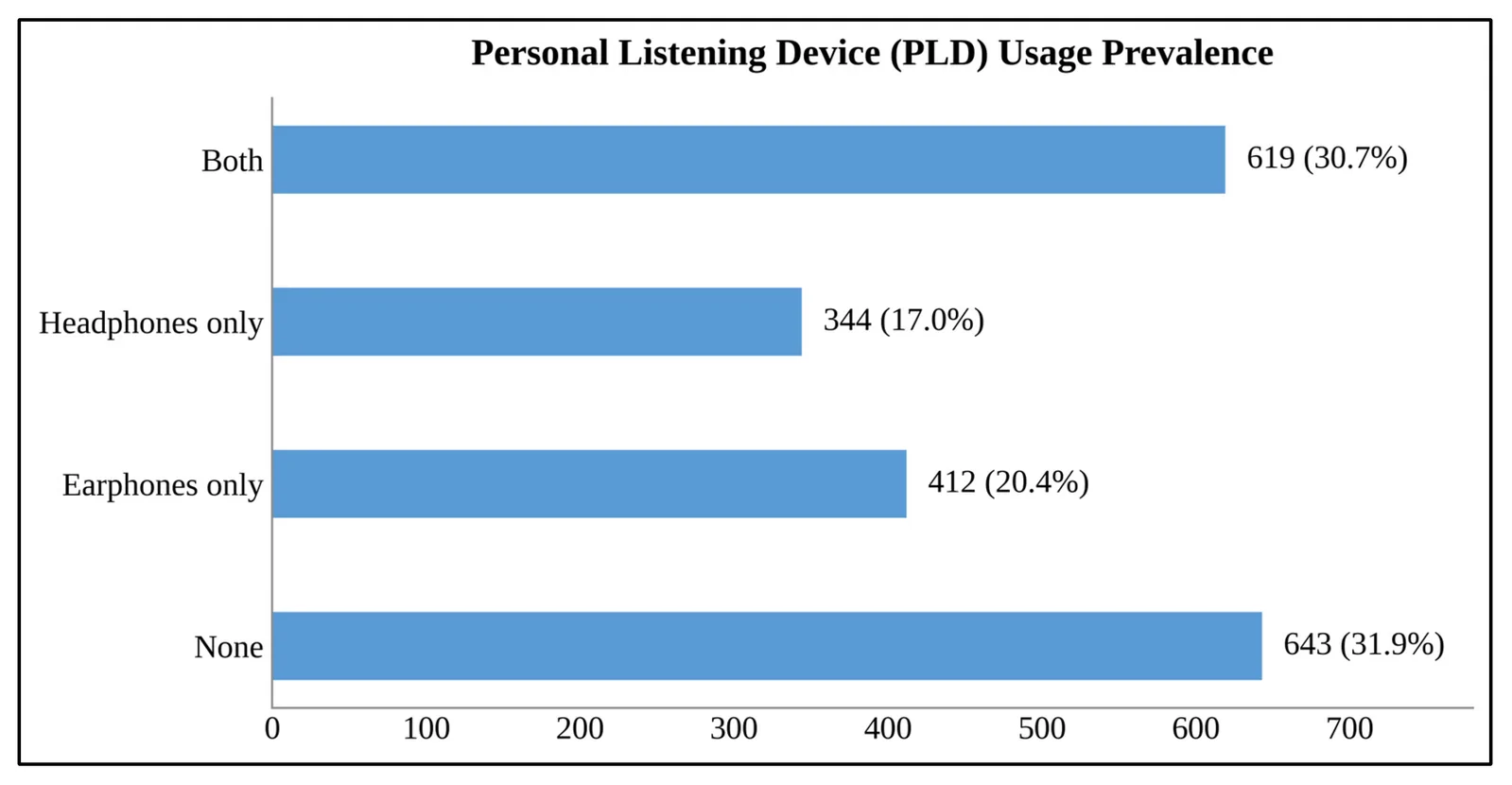
Distribution of personal listening device (PLD) usage categories among study participants (n = 2018).

Audio listening device usage patterns are presented in Table 3. Among all audio devices, earphones were the most used (n = 1031; 51.1%), followed by headphones (n = 963; 47.7%) and car audio systems (n = 587; 29.1%). When considering the single most frequently used device, earphones remained the predominant choice (n = 679; 33.6%), while 517 participants (25.6%) reported not using any audio listening device. With respect to personal listening devices (PLDs), defined as earphones and/or headphones, 643 participants (31.9%) reported no PLD use. Among PLD users (n = 1375), 619 (30.7%) used both earphones and headphones, 412 (20.4%) used earphones only, and 344 (17.0%) used headphones only (Figure 1).

### 3.4. Ear Health Status and Hearing Handicap

**Figure 2:**
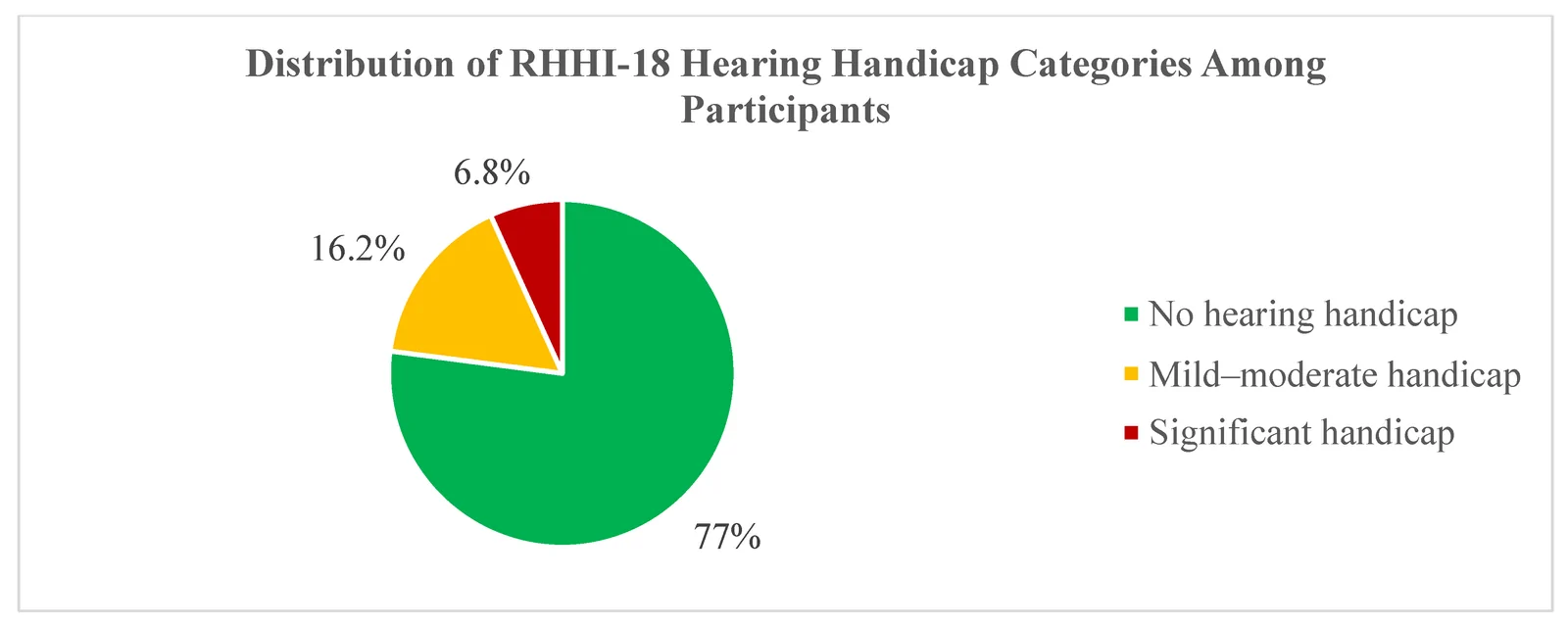
Distribution of RHHI-18 hearing handicap categories among study participants (n = 2018).

Ear health and hearing handicap outcomes for all study participants are presented in Table 4. Self-reported hearing loss was noted by 12.3% of participants (n = 248), while 87.7% (n = 1770) reported no hearing loss. The mean Revised Hearing Handicap Inventory (RHHI-18) total score was 10 ± 18 (range 0–72). The majority of participants demonstrated no hearing handicap (n = 1555; 77%), while 16.2% (n = 326) had mild-to-moderate handicap and 6.8% (n = 137) had significant handicap (Figure 2).

### 3.5. Multivariable Linear Regression: Predictors of RHHI-18 Score

The results of the multivariable linear regression analysis examining predictors of RHHI-18 score across all 2018 participants are presented in Table 5. Older age was independently associated with higher RHHI-18 scores (β = 0.16; 95% CI: 0.06 to 0.25; *p* = 0.002). Male sex was associated with significantly higher scores compared with females (β = 3.09; 95% CI: 1.38 to 4.80; *p* < 0.001), as was non-Saudi nationality compared with Saudi participants (β = 3.92; 95% CI: 0.78 to 7.05; *p* = 0.014).

Among medical conditions, stroke demonstrated the strongest association with RHHI-18 score (β = 21.51; 95% CI: 14.72 to 28.29; *p* < 0.001), followed by diabetes mellitus (β = 8.64; 95% CI: 5.50 to 11.79; *p* < 0.001) and hypertension (β = 4.84; 95% CI: 1.38 to 8.30; *p* = 0.006). All three categories of PLD use were independently associated with higher RHHI-18 scores relative to non-use: headphones only (β = 4.58; 95% CI: 2.26 to 6.89; *p* < 0.001), earphones only (β = 3.29; 95% CI: 1.19 to 5.40; *p* = 0.002), and combined use (β = 2.87; 95% CI: 0.88 to 4.85; *p* = 0.005). BMI, marital status, residence, educational level, income, smoking status, and physical activity were not significantly associated with RHHI-18 scores. The model explained 18.0% of the variance in RHHI-18 scores (R^2^ = 0.180; adjusted R^2^ = 0.166) and provided significantly better fit than an intercept-only model (AIC 17,043.5 vs. 17,363.8; F (22, 1995) = 17.94; likelihood-ratio χ^2^ (22) = 364.36; both *p* < 0.001). Analysis of variance confirmed independent associations for stroke, diabetes mellitus, hypertension, age, sex, nationality, personal listening device use, income, and residence (all *p* < 0.05).

## 4. Discussion

### 4.1. Principal Findings

This nationwide cross-sectional study of 2018 Saudi adults provides the largest assessment to date of PLD use and its association with perceived hearing handicap in the Kingdom. The principal findings are threefold. First, PLD use is highly prevalent, with more than two-thirds of participants (68.1%) reporting current use of earphones and/or headphones. Second, nearly one in four participants (23.0%) exhibited a measurable degree of functional hearing handicap as assessed by the RHHI-18. Third, multivariable regression identified all PLD use categories as independent predictors of higher hearing handicap scores, alongside demographic and clinical correlates including age, sex, nationality, stroke, diabetes mellitus, and hypertension.

### 4.2. Prevalence and Patterns of Personal Listening Device Use

The prevalence of earphone use (51.1%) and headphone use (47.7%) observed in this study is consistent with global trends in personal audio consumption. These figures are consistent with data from the Qassim region of Saudi Arabia, where Alenezi et al. reported that 94% of medical students used headphones [8], and with findings from India, where Saran et al. observed comparably high uptake among university students [9]. The predominance of earphone and headphone use in a young, predominantly student cohort suggests that PLD exposure is accumulating during a critical period of auditory development, heightening the long-term risk of hearing handicap. These trends are likely to intensify with the continued proliferation of wireless audio technology and media streaming platforms.

### 4.3. Prevalence of Hearing Handicap

Nearly one in four participants (23.0%; 16.2% mild-to-moderate and 6.8% significant) scored within the hearing handicap range of the RHHI-18, a substantial figure for a cohort with a mean age of 29 years. Interpreting this rate requires attention to what the instrument measures. The RHHI-18 captures perceived functional and emotional difficulty in everyday listening situations, a construct related to but distinct from audiometric threshold loss. National estimates of hearing impairment in Saudi Arabia, derived from clinical and audiometric case definitions, stand at approximately 1.4% [13]. The two figures therefore quantify different phenomena, and the difference between them is consistent with the established observation that perceived listening difficulty emerges earlier and is reported more frequently than measurable threshold shift. Prior Saudi work supports this reading, having documented high rates of otological complaints such as tinnitus and ear pain among device users in the Kingdom [14], symptoms that are commonly experienced well before audiometric criteria for hearing loss are met.

Comparison with international data reinforces this interpretation. Byeon reported a 22.6% prevalence of hearing loss among South Korean adolescents with prolonged earphone exposure [6], closely aligned with the rate observed here despite differences in population and measurement approach. By contrast, Dillard et al. recorded a considerably higher 48.8% prevalence of self-reported hearing difficulty using the RHHI in a large US cohort [11], a sample with a mean age of 63.7 years in which presbycusis would be expected to account for much of the elevation. Positioned between an adolescent and an older adult reference point, the rate observed in the present study indicates that perceived listening difficulty is already established rather than absent in young Saudi adults, which is the finding of principal clinical interest.

### 4.4. Association Between Personal Listening Device Use and Hearing Handicap

After controlling for demographic and clinical covariates, all three categories of PLD use were independently associated with higher RHHI-18 scores relative to non-users. Exclusive headphone use demonstrated the strongest association (β = 4.58; *p* < 0.001), followed by earphones only (β = 3.29; *p* = 0.002) and combined use (β = 2.87; *p* = 0.005). The larger coefficient observed for headphones compared with earphones may reflect the higher sustained acoustic energy delivered by over-ear devices, which enclose the pinna and create a sealed acoustic environment, thereby amplifying effective sound pressure levels at the eardrum. These findings contrast with those of Al-Qurashi et al., who reported that insert earphone users in Saudi Arabia had a higher prevalence of localized otological history compared with headphone users (32.4% vs. 23.7%) [10]. This apparent divergence is readily explained by outcome-measure differences: physical insertion trauma, cerumen impaction, and external ear canal inflammation are logically associated with in-ear devices, whereas over-ear headphones are more likely to produce sustained acoustic overexposure leading to functional auditory handicap as captured by the RHHI-18.

The dose–response relationship observed globally is corroborated by Byeon, who demonstrated that South Korean adolescents using earphones for more than 80 min daily faced a 4.7-fold increased risk of hearing loss and an 8.4-fold higher risk of subjective hearing problems [6]. Khoza-Shangase and Mokhethi similarly found that extended recreational device use was associated with perceived auditory difficulties among South African undergraduates [15]. These converging international findings reinforce the plausibility and generalizability of the association identified in the current study.

### 4.5. Sociodemographic and Clinical Predictors of Hearing Handicap

Beyond device use, several demographic and clinical predictors were independently associated with higher RHHI-18 scores. Older age (β = 0.16; *p* = 0.002) aligns with the well-established contribution of presbycusis to cumulative auditory dysfunction. Male sex was associated with significantly higher scores (β = 3.09; *p* < 0.001), consistent with evidence that males are more likely to engage in high-risk listening behaviors and to have occupational noise exposure [6]. Non-Saudi nationality was also a significant predictor (β = 3.92; *p* = 0.014), a finding that may reflect differential occupational noise exposures, healthcare access disparities, or language barriers to audiological care among expatriate workers.

The strong associations observed for stroke (β = 21.51; *p* < 0.001), diabetes mellitus (β = 8.64; *p* < 0.001), and hypertension (β = 4.84; *p* = 0.006) corroborate a well-established pathophysiological mechanism: vascular disease impairs cochlear microcirculation, accelerates auditory aging, and reduces the auditory cortex’s capacity to process degraded signals. These findings highlight that comorbidity management is inseparable from hearing health, and that patients with cardiometabolic conditions should be prioritized for audiological screening. Taken together, the covariates examined here accounted for 18.0% of the variance in RHHI-18 scores, indicating that perceived hearing handicap is shaped by a broader set of biological, environmental, and behavioral influences than those measured in this study.

### 4.6. Implications, Strengths, and Limitations

The findings of this study carry important implications for public health, clinical practice, and health policy in Saudi Arabia. The identification of PLD use as a potentially modifiable correlate of hearing handicap supports the need for population-level safe listening campaigns, particularly targeting young adults and students. Integration of the RHHI-18 as a brief screening tool within primary healthcare settings could enable early identification of at-risk individuals before irreversible auditory damage occurs. Device manufacturers should be encouraged to implement adaptive volume-limiting technologies and exposure-duration alerts. Future research should employ longitudinal designs to establish temporality and causality, incorporate objective audiometric assessments alongside self-report measures, and examine the impact of specific PLD features, including volume-limiting functions and active noise cancellation, on hearing outcomes. Characterizing residency status among non-Saudi participants would further refine interpretation of the nationality finding.

This study has several notable strengths. The large sample (n = 2018) spans multiple regions and age groups and provides broad geographical coverage of the adult population of the Kingdom. The use of the RHHI-18, a validated and psychometrically robust instrument, provides a standardized and clinically meaningful measure of hearing handicap. The multivariable regression approach adjusted for a broad range of demographic, clinical, and behavioral covariates. To our knowledge, this is the first nationwide study in Saudi Arabia to quantify PLD-related hearing handicap using a validated functional scale. Limitations include the cross-sectional design, which precludes causal inference. Recruitment through online platforms using convenience sampling limits the representativeness of the sample and may favor younger, more educated participants and frequent users of digital technology. Reliance on self-report is susceptible to recall and social desirability bias, and to cultural variation in the perception and reporting of hearing difficulty, and the absence of clinical audiometric data limits the ability to correlate subjective handicap with objective hearing thresholds. Characterization of PLD use was limited to device type, and associated variables such as volume levels, use duration, and other device features were not captured. Occupational noise exposure was not measured, which limits the ability to isolate the association between PLD use and hearing handicap independently of workplace noise. Genetic and hereditary determinants of hearing loss were not fully characterized. The Arabic version of the RHHI-18 was cross-culturally adapted for use in this study, but a full psychometric validation study in an Arabic-speaking population, including internal consistency, test–retest reliability, and confirmatory factor analysis against audiometric criteria, has not yet been conducted. Future studies incorporating audiometric evaluation and probability-based sampling designs will be essential to extend these findings.

## 5. Conclusions

Personal listening devices are now integral to daily life in Saudi Arabia, with more than two-thirds of adults reporting current use of earphones or headphones. This study demonstrates that PLD use is independently associated with perceived hearing handicap, with headphone use showing the strongest association. One in four participants exhibited measurable hearing handicap, occurring in a predominantly young cohort. Comorbid conditions, including stroke, diabetes mellitus, and hypertension, were also associated with higher scores. These findings underscore the need for targeted public health campaigns promoting safe listening practices, integration of standardized hearing handicap screening in primary care, and longitudinal research to establish causality. Protecting the hearing health of Saudi Arabia’s young population represents an achievable and time-sensitive public health priority.

## Figures and Tables

**Table 1:** Sociodemographic characteristics of the study participants (n = 2018).

Characteristics	Mean ± SD/Frequency (%)
Age (years)	29 ± 12
BMI (kg/m^2^)	24 ± 5.9
**Sex**	
Female	1089 (54.0%)
Male	929 (46.0%)
**Nationality**	
Saudi	1894 (93.9%)
Non-Saudi	124 (6.1%)
**Residence**	
Urban	1634 (81.0%)
Rural	384 (19.0%)
**Marital status**	
Single	1344 (66.6%)
Married	612 (30.3%)
Divorced/Widowed	62 (3.1%)
**Education level**	
Bachelor’s degree or diploma	1375 (68.1%)
High school or less	537 (26.6%)
Postgraduate degree	106 (5.3%)
**Monthly household income (SAR)**	
Less than 5000 (<~$1333)	553 (27.4%)
5000–9999 (~$1333–$2666)	450 (22.3%)
10,000–14,999 (~$2667–$3999)	447 (22.2%)
More than 15,000 (>~$4000)	568 (28.1%)

Abbreviations: n: sample size; SD: standard deviation; BMI: Body Mass Index; SAR: Saudi Arabian Riyal. USD equivalents are approximate, based on the exchange rate of 1 SAR = 0.2667 USD (1 USD = 3.75 SAR) as of 11 May 2026.

**Table 2:** Health conditions and lifestyle characteristics of the study participants (n = 2018).

Characteristics	Frequency (%)
**Chronic conditions**	
Diabetes mellitus	173 (8.6%)
Hypertension	142 (7.0%)
Hyperlipidemia	180 (8.9%)
Stroke	30 (1.5%)
Meniere’s disease	29 (1.4%)
Migraine	145 (7.2%)
Chronic headache	133 (6.6%)
History of ear procedure	240 (11.9%)
Congenital hearing loss	124 (6.1%)
Acoustic trauma	258 (12.8%)
Hearing aid use	94 (4.7%)
**Physical activity**	
No physical activity	802 (39.7%)
<30 min, 5 days per week	372 (18.4%)
≥30 min, 5 days per week	844 (41.8%)
**Smoking status**	
Never smoker	1647 (81.6%)
Current smoker	206 (10.2%)
Ex-smoker	165 (8.2%)

Abbreviations: n: sample size.

**Table 3:** Audio listening device usage patterns among study participants (n = 2018).

Characteristics	Frequency (%)
**Type of audio listening devices used (multiple responses)**	
Earphones	1031 (51.1%)
Headphones	963 (47.7%)
Car audio system	587 (29.1%)
Home speaker	234 (11.6%)
Portable speaker	172 (8.5%)
Hearing aid	94 (4.7%)
**Most frequently used audio listening device**	
None/do not use audio devices	517 (25.6%)
Earphones	679 (33.6%)
Headphones	484 (24.0%)
Car audio system	197 (9.8%)
Home speaker	66 (3.3%)
Portable speaker	64 (3.2%)
Hearing aid	11 (0.5%)

Abbreviations: n: sample size; PLDs: personal listening devices. a Percentages sum to more than 100% as participants could select multiple options. PLDs refer specifically to earphones and headphones used for personal audio listening.

**Table 4:** Ear health status and hearing handicap among study participants (n = 2018).

Characteristics	Mean ± SD/Frequency (%)
**Self-reported hearing loss**	
No	1770 (87.7%)
Yes	248 (12.3%)
**RHHI-18 total score**	10 ± 18

Abbreviations: n: sample size; SD: standard deviation; RHHI-18: Revised Hearing Handicap Inventory, 18-item version.

**Table 5:** Multivariable linear regression analysis of factors associated with RHHI-18 score (n = 2018).

Predictor	β	95% CI	*p*-Value
**Age (years)**	**0.16**	**0.06 to 0.25**	**0.002**
**BMI (kg/m^2^)**	0.02	−0.11 to 0.16	0.737
**Sex (reference: Female)**			
*Male*	**3.09**	**1.38 to 4.80**	**<0.001**
**Nationality (reference: Saudi)**			
*Non-Saudi*	**3.92**	**0.78 to 7.05**	**0.014**
**Marital status (reference: Single)**			
*Married*	−0.86	−3.36 to 1.64	0.501
*Divorced/Widowed*	1.24	−3.46 to 5.93	0.606
**Residence (reference: Rural)**			
*Urban*	1.37	−0.61 to 3.35	0.174
**Education level (reference: High school or less)**			
*Bachelor’s degree or diploma*	1.20	−0.52 to 2.91	0.171
*Postgraduate degree*	1.04	−2.62 to 4.70	0.578
**Monthly household income (reference: Below 5000 SAR)**			
*5000–9999 SAR*	1.66	−0.47 to 3.78	0.126
*10,000–14,999 SAR*	1.17	−1.04 to 3.38	0.298
*Above 15,000 SAR*	−1.10	−3.14 to 0.95	0.294
**Medical conditions (reference: Absent)**			
*Diabetes mellitus*	**8.64**	**5.50 to 11.79**	**<0.001**
*Hypertension*	**4.84**	**1.38 to 8.30**	**0.006**
*Stroke*	**21.51**	**14.72 to 28.29**	**<0.001**
**Smoking status (reference: Never smoker)**			
*Current smoker*	1.07	−1.50 to 3.63	0.414
*Ex-smoker*	−2.03	−4.79 to 0.72	0.148
**Physical activity (reference: No physical activity)**			
*<30 min, 5 days/week*	−0.98	−3.02 to 1.06	0.346
*≥30 min, 5 days/week*	0.72	−0.92 to 2.35	0.392
**PLDs usage (reference: No PLDs use)**			
*Earphones only*	**3.29**	**1.19 to 5.40**	**0.002**
*Headphones only*	**4.58**	**2.26 to 6.89**	**<0.001**
*Both earphones and headphones*	**2.87**	**0.88 to 4.85**	**0.005**

R^2^ = 0.180; Adjusted R^2^ = 0.166. Abbreviations: β: unstandardized regression coefficient; CI: confidence interval; BMI: Body Mass Index; PLDs: personal listening devices; SAR: Saudi Arabian Riyal. Bold rows indicate statistically significant associations (*p* < 0.05).

## Data Availability

The datasets generated and analyzed during the current study are available from the corresponding author upon reasonable request. Data are not publicly available due to informed consent restrictions and the risk of participant re-identification.

## Abbreviations

The following abbreviations are used in this manuscript:

BMI	Body Mass Index
CI	Confidence Interval
PLDs	Personal Listening Devices
RHHI-18	Revised Hearing Handicap Inventory, 18-item version
SAR	Saudi Arabian Riyal
SD	Standard Deviation
STROBE	Strengthening the Reporting of Observational Studies in Epidemiology
UNHS	Universal Newborn Hearing Screening
USD	United States Dollar
